# Protective Effect of *Ginkgo biloba* and Magnetized Water on Nephropathy in Induced Type 2 Diabetes in Rat

**DOI:** 10.1155/2018/1785614

**Published:** 2018-06-11

**Authors:** Ahmed E. Zayed, Ahmed Saleh, Asmaa M. S. Gomaa, Mahmoud Abd-Elkareem, Mamdouh M. Anwar, Khaled M. A. Hassanein, Mohsen M. Elsherbiny, Ahmed M. Kotb

**Affiliations:** ^1^Department of Anatomy and Histology, Faculty of Veterinary Medicine, Assiut University, Assiut 71515, Egypt; ^2^Department of Biology, Faculty of Science, Jazan University, Jazan, Saudi Arabia; ^3^Exploratory Center of Science and Technology, Cairo, Egypt; ^4^Department of Physics, Faculty of Science, Jazan University, Jazan, Saudi Arabia; ^5^Medical Physiology Department, Faculty of Medicine, Assiut University, Assiut, Egypt; ^6^Department of Pharmacology, Faculty of Pharmacy, Jazan University, Jazan, Saudi Arabia; ^7^Department of Pathology and Clinical Pathology, Faculty of Veterinary Medicine, Assiut University, Assiut 71526, Egypt; ^8^Deanship of Scientific Research, Jazan University, Jazan, Saudi Arabia; ^9^College of Public and Tropical Medicine, Jazan University, Jazan, Saudi Arabia

## Abstract

We aimed in our current study to explore the protective effect of *Ginkgo biloba* (GB) and magnetized water (MW) against nephrotoxicity associating induced type 2 diabetes mellitus in rat. Here, we induced diabetes by feeding our lab rats on a high fat-containing diet (4 weeks) and after that injecting them with streptozotocin (STZ). We randomly divided forty rats into four different groups: nontreated control (Ctrl), nontreated diabetic (Diabetic), Diabetic+GB (4-week treatment), and Diabetic+MW (4-week treatment). After the experiment was finished, serum and kidney tissue samples were gathered. Blood levels of glucose, triglycerides, cholesterol, creatinine, and urea were markedly elevated in the diabetic group than in the control group. In all animals treated with GB and MW, the levels of urea, creatinine, and glucose were significantly reduced (all *P* < 0.01). GB and MW attenuated glomerular and tubular injury as well as the histological score. Furthermore, they normalized the contents of glutathione reductase and SOD2. In summary, our data showed that GB and MW treatment protected type 2 diabetic rat kidneys from nephrotoxic damages by reducing the hyperlipidemia, uremia, oxidative stress, and renal dysfunction.

## 1. Introduction

Diabetes mellitus (DM) is known to be a metabolic disorder chronic disease resulting from variable interactions of hereditary and environmental factors. DM is characterized by abnormalities in the insulin metabolism with subsequent disorders in carbohydrates, proteins, and fat metabolism. Furthermore, DM is usually associated with kidney damage [1]. It is well known that DM is subdivided into DM type 1 and DM type 2. The main cause of DM type 1 is the destruction of the pancreatic *β*-cells leading to deficiency of insulin production and secretion and that usually comes with hyperglycemia and ketoacidosis [2]. DM type 2 is known to be more prevalent; its main cause is obesity and characterized by hyperlipidemia and hyperinsulinemia [3].

Free radicals have pathogenic effects and complications of diabetes. Certainly, reactive oxygen species (ROS) formation is the shortest result of hyperglycemia [4]. Disturbances in antioxidant defense systems and ROS encourage the accumulation of renal oxidative stress in diabetic patients [5]. Therefore, being a substance with ROS scavenging ability, this substance then obtained a possible efficiency on the patients suffering from diabetes accompanied with excessive oxidative stress level [6]. Antioxidant defense mechanisms involve both nonenzymatic and enzymatic strategies. The most mutual antioxidant enzymes are glutathione reductase and superoxide dismutase [7].

*Ginkgo biloba* is a native tree in East Asia. Its extract is used frequently in many medicine recipes. The *Ginkgo biloba* leaf extract consists mainly of terpenoids and glycosides which have antioxidant potency [8]. *Gingko biloba* extracts showed significant effects on the whole antioxidant status of the organs especially on glutathione peroxidase and superoxide dismutase [9]. Moreover, it has been shown that *Ginkgo biloba* was able to rescue the cardiac phenotype in streptozotocin-induced diabetic rats [10]. Furthermore, *Ginkgo biloba* has a vascular rescuing effect as well as an antiapoptotic effect [11–13]. In addition, it has been shown that *Ginkgo biloba* could rescue renal injury in brain death induced-nephrotoxicity [14] and adriamycin-induced hyperlipidemic nephrotoxicity [15] and in uranium-treated mice [16].

It has been shown recently that magnetized water has an efficient antioxidant effect and has a high ability to diffuse rabidly into tissues. In addition, it has been reported that one of the major roles of magnetic water is its antiapoptotic renal effect [17–20]. It has also been shown that magnetized water administration can reduce blood glucose level and improve the antioxidant status and lipid profiles in the heart, spleen, and lung of streptozotocin-induced diabetic rats [19]. Furthermore, magnetized water has been used to rescue cisplatin-induced nephrotoxicity [21, 22], ferric-induced nephrotoxicity [23], and gentamicin-induced nephrotoxicity [24]. Even more, drinking magnetic water may have a useful role in inhibiting blood parameter disorders of type 2 diabetes mellitus [25]. Moreover, magnetized water is believed to have an antioxidant effect which could be a result of the increase in glutathione peroxidase concentration in serum [26].

Lack of information about kidney disorders accompanying induced type 2 diabetes and how to rescue these disorders prompted us to use a diabetic rat model and try to rescue its renal disorders. Our novel study described STZ-induced nephrotoxicity (DM type 2) and how to ameliorate the nephrotoxic effect using the extract of *Ginkgo biloba* leaves as well as magnetic water.

## 2. Material and Methods

### 2.1. Experimental Animals

**Figure 1:**
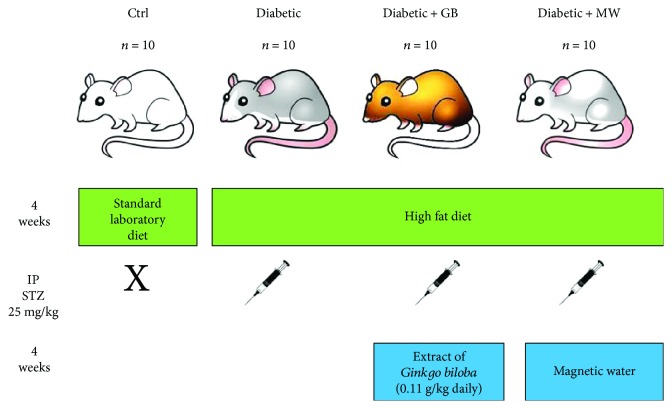
Experimental scheme. Scheme of our experimental groups, number of rats we used, the treatment way, and duration. GB: *Ginkgo biloba*; MW: magnetized water; IP STZ: intraperitoneal injection of streptozotocin.

Forty adult Wistar rats (male) (weigh: 200 ± 10 g, age: 3.5 months) were obtained from an animal house in Jazan University, KSA. The rats were kept in standard plastic cages under standard environmental conditions. Rats were divided equally into four groups. The different rat groups were treated as depicted in Figure 1.

STZ (Sigma Chemical Co., St. Louis, MO, USA) and *Ginkgo biloba* leaf extract (KARA, d. d., Novo mesto company, Slovenia) were used and dissolved in water.

### 2.2. Magnetic Water Preparation

Magnetized water was prepared by passing drinking water through our handmade electromagnet unit. Water was pumped through a flexible tube by a water pump installed inside the unit. The produced magnetic strength was 600 Gauss (measured by a WT10A Tesla meter), which is an average value of the magnetic field strength used in experimental animals and proved to have no pathological effect [27]. The used magnetic field was uniform and perpendicular to the water flow. Water flow was at a relatively low speed (0.34 l/minute) to avoid overflow.

### 2.3. Blood Indices

Serum levels (*n* = 10) of total cholesterol value (TC), triglyceride value (TG), high-density lipoprotein value (HDL), low-density lipoprotein value (LDL), urea, and creatinine were measured spectrophotometrically, using colorimetric assay kits. Low-density lipids (LDL-C) were calculated using Friedewald's formula [28].

### 2.4. Immunohistology

For paraffin tissue sections, kidneys of scarified rats (*n* = 10) were collected and fixed in 4% neutral buffered formalin. Fixed kidneys were dehydrated in ascending grades of ethanol, cleared in methyl benzoate, and then embedded in paraffin wax. Paraffin blocks were sectioned at 5 *μ*m thickness. Sections were stained with hematoxylin and eosin (H&E) (Sigma-Aldrich).

The following antibodies were used according to previously published protocols [29]: anti-glutathione reductase and anti-SOD2 (Chongqing Biospes Co., Ltd, China). HRP DAB Kit (Genemed Biotechnologies). ImageJ software was used for histological sections analysis.

### 2.5. Semithin Sections

2 mm thick kidney samples were fixed and stained with Toluidine Blue according to Kotb et al. [30]. Semithin sections were examined, and light was photographed microscopically.

### 2.6. Statistical Analysis

Analysis of data was done by means of one-way ANOVA. Data were presented as mean ± SE. Student's *t*-test was used during these statistical analyses. Differences were significant when *P* < 0.05 (^∗^*P* ≤ 0.05, ^∗∗^*P* ≤ 0.01, and ^∗∗∗^*P* ≤ 0.001).

### 2.7. Ethical Statement

All experiments were performed in Jazan University, KSA, and according to their laws.

## 3. Results

### 3.1. GB and MW Protect the Glomerular Structure against Induced Type 2 Diabetic Nephrotoxicity

**Figure 2:**
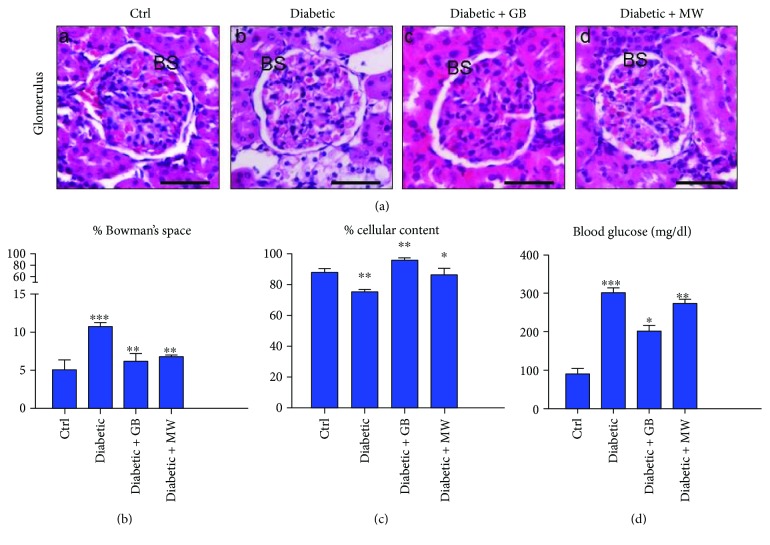
GB- and MW-protective effects against diabetic nephropathy. (a) Paraffin tissue sections stained against H&E. Dilated Bowman's space (BS) in diabetic glomeruli comparable to control in Diabetic+GB and Diabetic+MW. Scale bar: 50 *μ*m. Image size measurements (using ImageJ software) of Bowman's space and cellular contents in relation to the whole glomerulus size. % size of Bowman's space was markedly increased in diabetic glomeruli and decreased gradually after the use of GB and MW (b). In diabetic glomeruli, % size of cellular content was decreased and then increased with GB and MW administration (c). Blood glucose level was increased compared to the control in diabetic rat and then decreased after the use of GB and MW (d).

To confirm the efficiency of our induced nephrotoxic rat model (Figure 1), we performed blood glucose analysis. We found that the blood glucose level in diabetic rats (301 mg/dl) was increased comparable with the control (90 mg/dl) (Figure 2, D).

In order to investigate the outcome of induced type 2 diabetes on the rat glomerular structure, we stained paraffin sections with H&E. Interestingly, structural changes in the glomeruli were induced in diabetic rats compared with the control (Ctrl). Bowman's space size and glomerular cellular contents combined directly with the glomerular healthy condition. We found a marked dilatation of Bowman's space (BS) in diabetic rats compared with Ctrl (Figure 2(a), A and B). In addition, a decrease in glomerular cellular contents in diabetic rats was found (Figure 2(a), A and B). Using ImageJ software, the size of different parts of the glomerulus in relation to the whole glomerulus size was measured (Figures 2(b) and 2(c)). Bowman's space size in diabetic glomeruli was doubled (10%) compared with Ctrl (5%) (Figure 2(b)). In addition, the cellular content size in diabetic glomeruli was decreased compared with Ctrl, 75% and 86%, respectively (Figure 2(c)).

The protective effect of GB and MW on the kidney was studied. We provided diabetic rats with GB and MW, and then we showed a rescue effect on the glomerular phenotype by GB and MW treatment. Also, the glomerular structures (Bowman's space and the cellular content) were nearly comparable to control after GB and MW treatment (Figure 2(a), A, C, D). Size changes of Bowman's space and cellular contents were determined with ImageJ; we showed significant variations in GB- and MW-treated glomeruli compared with Ctrl. After GB and MW treatment, we found a significant decrease in the blood glucose level compared with diabetic rats at 203, 273, and 301 mg/dl, respectively (Figure 2(d)).

### 3.2. GB and MW Ameliorate Diabetic Effects on the Glomerular Vessels

**Figure 3:**
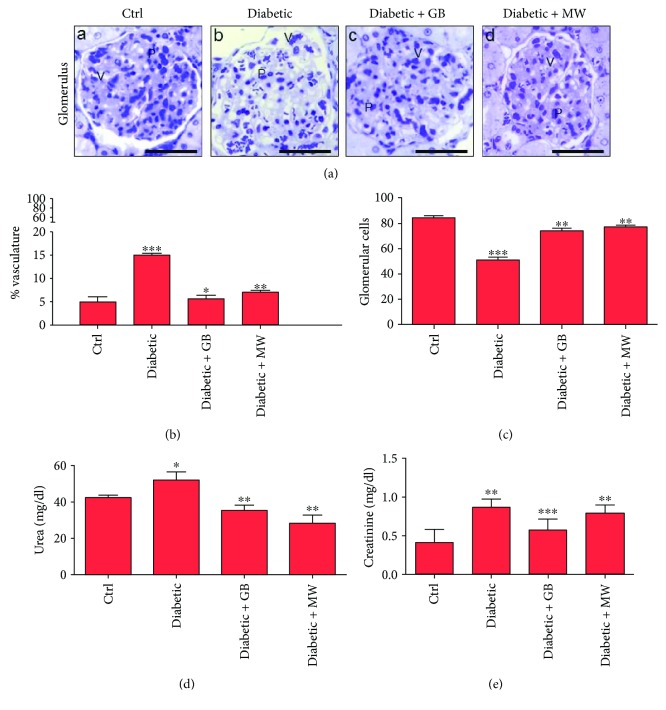
In diabetic glomeruli, disturbances in vascular size and podocyte number were rescued after GB and MW treatment. ((a), A–D) Semithin sections stained with Toluidine Blue. Dilated glomerular vessels and less podocytes in diabetic glomeruli compared with Ctrl. Glomerular vessel size (V) and podocyte number (P) in Diabetic+GB and Diabetic+MW were comparable to control glomeruli. Scale bar: 50 *μ*m. ImageJ analysis displayed a marked vascular dilatation (*P* < 0.05) in diabetic glomeruli compared to Ctrl. Glomerular vessel diameter was decreased after GB and MW treatment (b). In diabetic glomeruli, ImageJ analysis presented a decrease in % podocyte number compared to Ctrl (c). On the other hand, % podocyte number increased after the use of GB and MW (c). In diabetic rats, levels of blood urea and creatinine were decreased after the use of GB and MW (d, e).

To confirm the toxic effect of diabetes on the vessels of glomeruli, semithin sections stained with Toluidine Blue were analyzed. We found that diabetic glomeruli had dilated vessels compared to Ctrl (Figure 3(a), A and B). The correlation between glomerular vessel size and the whole glomerulus size was measured (Figure 3(b)). In diabetic glomeruli, a significant vascular dilatation (*P* < 0.05) compared to the control was determined (15% and 5%, resp.) (Figure 3(b)).

Podocytes surrounded the glomerular blood vessels and interdigitate together, forming a mesh of filtrating slits. Concomitantly with decreased size of glomerular content in diabetic glomeruli (Figure 2(c)), we also found a decrease in the number of podocytes compared with Ctrl (Figure 3(a), A and B). ImageJ counting analysis showed a significant decrease in podocyte number in diabetic glomeruli compared to Ctrl (51% and 84%, resp.) (Figure 3(c)).

Furthermore, we found that serum creatinine and urea levels were ominously increased (*P* < 0.05) in rats suffering from diabetes compared to control (Figures 3(d) and 3(e)).

To assess GB- and MW-protective effects on diabetic glomeruli, we provided the diabetic rats with GB and MW (Figure 1). We found that vascular size variations of GB- and MW-treated glomeruli were markedly decreased than those of diabetic glomeruli (Figure 3(a), B–D). The diameter of dilated glomerular vessels was significantly decreased in diabetic glomerular vessels after GB and MW treatment (15, 6, and 7% of the whole glomerulus size) (Figure 3(b)).

In concomitance with increased glomerular cellular contents in GB- and MW-treated glomeruli (Figure 2(a), (C, D)), an increased podocyte number in GB and MW glomeruli was found (Figure 3(c)).

Moreover, comparable with diabetic rats, we observed that serum creatinine and urea levels were significantly decreased (*P* < 0.05) after GB and MW treatment (Figures 3(d) and 3(e)).

### 3.3. GB and MW Treatments Ameliorate the Destructive Diabetic Effect on Renal Tubules

**Figure 4:**
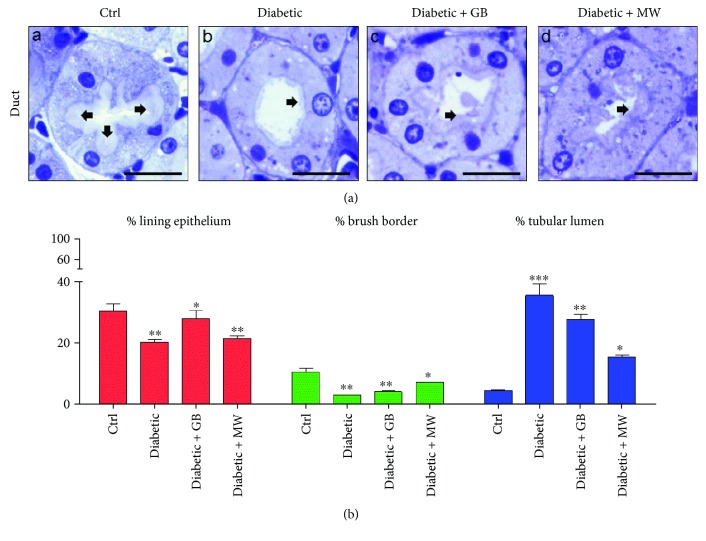
GB- and MW-protective effects on the renal tubules against induced type 2 diabetic nephrotoxicity. ((a), A–D) Toluidine Blue-stained semithin sections. The diabetic renal tubular epithelium increased in thickness compared with Ctrl. Epithelium thickness decreased after the treatment with GB and MW. Furthermore, the brush border thickness (arrow) in the diabetic renal tubule is rescued after the treatment with GB and MW. In addition, the dilated diabetic tubules became comparable to the control after the administration of GB and MW. Scale bar: 20 *μ*m. Image analysis confirmed thickness disturbances of the lining epithelium, brush border, and lumen of the diabetic renal tubules and the rescue effect of the use of GB and MW (b).

To examine the diabetic effects on renal tubules, we used semithin sections stained with Toluidine Blue. There is a marked decrease in the tubular lining epithelium and the brush border thicknesses compared to Ctrl (Figure 4(a), A and B). In addition, we noticed a marked increase in the tubular lumen in diabetic rats compared with Ctrl (Figure 4(a), A and B). ImageJ enabled us to measure the correlation ratio between the whole tubule and the thickness of the lining epithelium, brush border, and tubular lumen. We found a significant decrease in the thickness of the lining epithelium and brush border of diabetic rats (20% and 3%, resp.) compared to Ctrl (30.1% and 10.5%, resp.) (Figure 4(b)). Furthermore, the tubular lumen of diabetic rats was markedly dilated compared to the control (35.5% and 4.5%, resp.) (Figure 4(b)).

Interestingly, the tubular lining epithelium and brush border thicknesses decreased after GB and MW treatment compared to diabetic animals (Figure 4(a), B–D). Furthermore, ImageJ analysis showed that the treatment with GB and MW was able to rescue the tubular structural disturbances which occurred as a result of induced diabetes. We also noticed that the tubular lining epithelium thickness was significantly increased after GB and MW treatment compared to diabetic rats (4%, 7%, and 3%, resp.) (Figure 4(b)).

Moreover, we observed that the thickness of the tubular brush border was significantly increased after GB and MW treatment compared to the case in diabetic animals (28%, 21%, and 20%, resp.) (Figure 4(b)). Additionally, the tubular lumen after GB and MW treatment was decreased in size compared to diabetic animals (27.7%, 15.3%, and 35.5%, resp.).

Altogether, GB and MW treatment was able to ameliorate the damaging effect of induced type 2 diabetes on the renal tubular system.

### 3.4. GB and MW Treatment Weakened the Diabetic Effect on the Glomerular Glutathione Reductase and SOD2 Protein Expressions

To investigate glomerular oxidative stress after type 2 diabetes induction, we stained against glutathione reductase and SOD2 antibodies.

**Figure 5:**
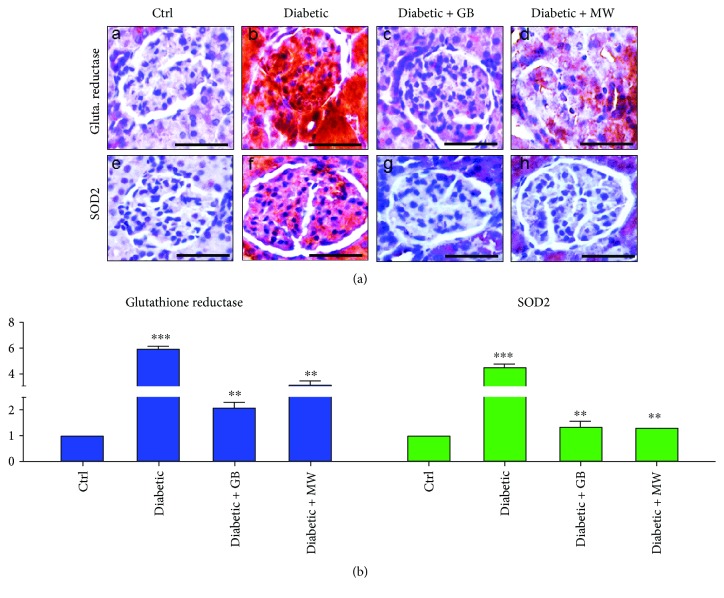
GB- and MW-protective effect against oxidative stress induced by type 2 diabetes. ((a), A–D) Paraffin sections stained with antiglutathione reductase antibody. The expression of glutathione reductase was increased in dabetic glomeruli and back to almost normal after the use of GB and MW. Furthermore, ((a), E–H) paraffin tissue sections stained with anti-SOD2 antibody. SOD2 expression was intensively increased in diabetic glomeruli with the use of GB and MW comparable with the control. Scale bar: 50 *μ*m. ImageJ analysis displayed a significant increase in glutathione reductase and SOD2 intensities in diabetic glomeruli compared to Ctrl. In Diabetic+GB and Diabetic+MW glomeruli, glutathione reductase and SOD2 protein expression intensities were decreased to be comparable with Ctrl (b).

In diabetic glomeruli, we found a marked increase in glutathione reductase and SOD2 expressions compared with the control (Figure 5(a), A and B, E and F, resp.).

ImageJ analysis showed that glutathione reductase expression intensity was significantly increased up to 6-fold in diabetic glomeruli compared with Ctrl (Figure 5(b)). Moreover, in diabetic glomeruli, we noticed a significant increase in SOD2 intensity up to 4.5-fold compared with the control (Figure 5(b)).

Interestingly, glutathione reductase and SOD2 protein expressions were comparable to control after GB and MW treatment (Figure 5(a)). ImageJ analysis confirmed the reduction in the intensity of glutathione reductase and SOD2 after GB and MW treatment (Figure 5(b)).

Altogether, GB and MW treatments were able to reduce renal oxidative stress in diabetic glomeruli.

### 3.5. GB and MW Treatment Ameliorate High Levels of Blood Triglycerides and Cholesterol in Type 2 Diabetes

Cholesterol is a vital lipid component important for the proper body function and normal cellular metabolism. An important type of fat that the body uses for storing energy is triglycerides. However, high blood fatty contents (cholesterol and triglyceride) are a risk factor that increases the chance of getting diseases like diabetes, heart disease, and atherosclerosis. To investigate whether induced type 2 diabetes affects the level of blood fatty profiles, total blood cholesterol and triglyceride levels were measured.

**Figure 6:**
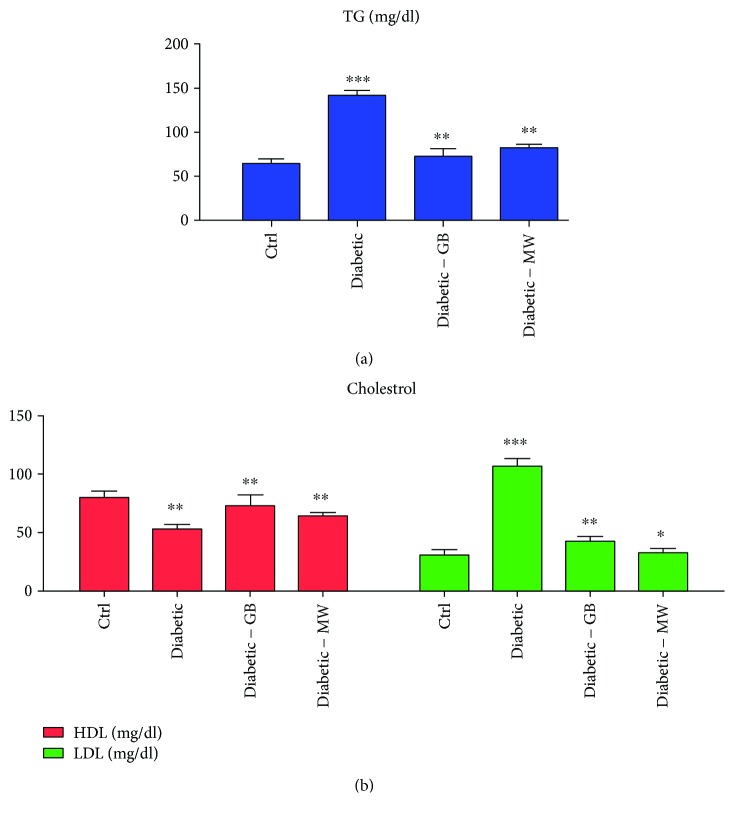
Lipid profiling of diabetic rats before and after GB and MW treatment. (a) and (b) showed triglycerides and cholesterol (LDL, HDL), respectively. The results reveal that a 4-week treatment with GB and MW remarkably normalized to some extent the circulating cholesterols and triglycerides in induced diabetic rats. Bars are means ± SEM (*n* = 5).

We also observed high fatty substances (hypercholesterolemia and high triglyceride) in diabetic rats compared with Ctrl (Figure 6(a)). Remarkably, in diabetic rats the cholesterol and triglyceride levels were decreased to almost normal after GB and MW treatments (Figure 6(a)).

There are two kinds of cholesterol: a good (HDL: high-density lipoproteins) and a bad (LDL: low-density lipoproteins) cholesterol. The increase in LDL caused many kinds of diseases. We found that HDL was decreased in diabetic rats compared with the Ctrl (Figure 6(b)). In contrast, LDL was increased in diabetic rats and cause hypercholesterolemia in these rats compared with Ctrl (Figure 6(b)).

To investigate the rescue ability of GB and MW on the high blood fatty contents in type 2 diabetic rats, we treated these diabetic rats with GB and MW, then we measured both cholesterol forms (HDL and LDL) (Figure 6(b)). We noticed an HDL increase and LDL decrease after GB and MW treatment compared to nontreated diabetic rats (Figure 6(b)).

Altogether, hypercholesterolemia in diabetic rats is mainly caused by increased LDL and triglycerides in rats. GB and MW treatment enabled us to ameliorate the nephrotoxic effect on the blood parameters in diabetic rats.

## 4. Discussion

Our current study displays that nephrotoxicity induced by diabetes could be rescued by the treatment with *Ginkgo biloba* extract and magnetized water. Likewise, a concomitant reduction in nephrotoxic effects after *Ginkgo biloba* treatment was mentioned before [14–16, 31–34] as well as magnetized water treatment [19, 21, 25–27].

In agreement with a previous study [35], we recognized that diabetes-induced nephrotoxicity in rats was categorized by blood parameter disturbances, specifically parameters important for kidney functions like urea, cholesterol, glucose, and creatinine levels.

Glomerular cell loss is considered to be a result of diabetes-induced nephrotoxicity [36]. Furthermore, diabetes-induced nephrotoxicity affects the blood vessel diameter, a vasodilatation effect [37]. Glomerular vasodilatation could induce a podocyte mechanical stretch leading to foot process effacement and, after that, cellular detachment. In agreement to what has been published before [38–40], podocyte stretch induces a decreased podocyte nephrin expression, the main slit diaphragm protein, leading to disturbances in the glomerular filtration function and proteinuria.

Furthermore, accumulation of edematous fluids in diabetic patients induces an increase in Bowman's space. The two main causes of glomerular edema formation are the increased movement of renal fluid from the glomerular pool to the urinary pool and blockade of the renal tubular system. As described before [41, 42], dilated glomerular vessels could increase the vascular endothelium fenestrae leading to increased fluid movements from the glomerular pool to the urinary pool inducing edema formation. In agreement to what was previously mentioned [43], we conclude that the induced oxidative stress could induce a lot of cellular destruction and accumulation of cellular depresses and in turn obstruct the renal tubular system.

Moreover, we found that type 2 diabetes has an effect on the renal tubule structure; it induces a marked decrease in the tubular brush border thickness leading to a case of standing renal fluid and dilatation of the lumen of renal tubules. On the other hand, the renal tubular structural abnormalities could disturb the normal fluid uptake leading to proteinuria. In the current study, not only was the renal structure disturbed after diabetes induction but also the protein expression pattern was markedly deviated than the normal.

In water, it was hypothesized that the magnetic field induces changes in the hydrogen protons by magnetic resonance and the H-bond gets distorted and weaker, affecting hydration and protonation of ions, and therefore, hydrogen-rich water can scavenge ROS [6, 44].

In agreement to other studies [45], we found that diabetic kidney shows an elevated level of oxidative stress marker like glutathione reductase and SOD2. Increased oxidative stress was considered to be an inflammatory and apoptotic trigger leading to cell loss [1, 6, 46].

It has been mentioned that the best metabolic control could stop the diabetic renal injury [47]. The protection can be done by several ways that include catalyzing free radical scavenging by intracellular antioxidant enzymes [48].

Absence of *Ginkgo biloba* hazard effects [49], low effect of high kidney related-serum parameters [50, 51], renal injury attenuating effect [14–16], antioxidant effect [52] and fatty acid regulation effect [53–55], encouraged us to use it to decrease nephrotoxic effects in diabetic rats.

On the other hand, the typical structure of the water molecule will have similar qualities of a magnetic pole, where the oxygen is slightly negative and the hydrogen is slightly positive [38, 39]. It is well known that the surface tension of water could be reduced by the magnetic field, making it softer, thinner, and absorbable [56]. In addition, it was hypothesized that the magnetic field alters the magnetic spin of hydrogen protons in water by magnetic resonance and the H-bond gets distorted and weaker, affecting hydration and protonation of ions, and therefore, the hydrogen-rich water can scavenge ROS [44, 57]. It has been also postulated that the magnetized water improves oxidative stress [18, 19, 48]. All previously mentioned advantages of magnetic water prompted us to use it to avoid the nephrotoxic effect of induced diabetes in rats.

In conclusion, our novel results showed that *Ginkgo biloba* and magnetized water could protect the kidney against diabetic nephropathy. *Ginkgo biloba* and magnetized water treatment have a rescue effect on the diabetic hazard effects on the glomerular Bowman space, glomerular vessels, and podocytes.

Our studies present a promising use for *Ginkgo biloba* and magnetized water in the treatment of diabetes mellitus type 2.

## Data Availability

The data used to support the findings of this study are available from the corresponding author upon request.

## Abbreviations

STZ: Streptozotocin
MG: Magnetized water
GB: *Ginkgo biloba*
DM: Diabetes mellitus
ROS: Reactive oxygen species
HDL: High-density lipoproteins
LDL: Low-density lipoproteins.
