# Corrigendum to “Protective Effect of *Ginkgo biloba* and Magnetized Water on Nephropathy in Induced Type 2 Diabetes in Rat”

**DOI:** 10.1155/2018/1094650

**Published:** 2018-08-09

**Authors:** Ahmed E. Zayed, Ahmed Saleh, Asmaa M. S. Gomaa, Mahmoud Abd-Elkareem, Mamdouh M. Anwar, Khaled M. A. Hassanein, Mohsen M. Elsherbiny, Ahmed M. Kotb

**Affiliations:** ^1^Department of Anatomy and Histology, Faculty of Veterinary Medicine, Assiut University, Assiut 71515, Egypt; ^2^Department of Biology, Faculty of Science, Jazan University, Jazan, Saudi Arabia; ^3^Exploratory Center of Science and Technology, Cairo, Egypt; ^4^Department of Physics, Faculty of Science, Jazan University, Jazan, Saudi Arabia; ^5^Medical Physiology Department, Faculty of Medicine, Assiut University, Assiut, Egypt; ^6^Department of Pharmacology, Faculty of Pharmacy, Jazan University, Jazan, Saudi Arabia; ^7^Department of Pathology and Clinical Pathology, Faculty of Veterinary Medicine, Assiut University, Assiut 71526, Egypt; ^8^Deanship of Scientific Research, Jazan University, Jazan, Saudi Arabia; ^9^College of Public and Tropical Medicine, Jazan University, Jazan, Saudi Arabia; ^10^Department of Anatomy and Cell Biology, University Medicine Greifswald, Greifswald, Germany

In the article titled “Protective Effect of *Ginkgo biloba* and Magnetized Water on Nephropathy in Induced Type 2 Diabetes in Rat” [1], there was a missing affiliation for the last author. The corrected authors' list and affiliations are shown above.
